# Ductular reaction-on-a-chip: Microfluidic co-cultures to study stem cell fate selection during liver injury

**DOI:** 10.1038/srep36077

**Published:** 2016-10-31

**Authors:** Amranul Haque, Pantea Gheibi, Gulnaz Stybayeva, Yandong Gao, Natalie Torok, Alexander Revzin

**Affiliations:** 1 Department of Biomedical Engineering, University of California Davis, CA 95616, USA; 2 Department of Internal Medicine, University of California Davis, Sacramento, CA 95835, USA

## Abstract

Liver injury modulates local microenvironment, triggering production of signals that instruct stem cell fate choices. In this study, we employed a microfluidic co-culture system to recreate important interactions in the liver stem cell niche, those between adult hepatocytes and liver progenitor cells (LPCs). We demonstrate that pluripotent stem cell-derived LPCs choose hepatic fate when cultured next to healthy hepatocytes but begin biliary differentiation program when co-cultured with injured hepatocytes. We connect this fate selection to skewing in production of hepatocyte growth factor (HGF) and transforming growth factor (TGF)-β1 caused by injury. Significantly, biliary fate selection of LPCs was not observed in the absence of hepatocytes nor did it happen in the presence of TGF-β inhibitors. Our study demonstrates that microfluidic culture systems may offer an interesting new tool for dissecting cellular interactions leading to aberrant stem cell differentiation during injury.

The liver is known for its regenerative capacity driven by proliferation of adult hepatocytes^1^. However, chronic chemical injury, for example with ethanol, damages the liver and impedes proliferation of hepatocytes. In such scenarios, regenerative processes are thought to be driven by liver progenitor cells – bi-potent adult stem cells capable of differentiating into either hepatocytes or biliary epithelial cells (cholangiocytes)^1^,^2^,^3^. Clinical evidence points to increase in the number of liver cells expressing progenitor cell markers in patients with chronic liver disease, including alcoholic hepatitis^4^. Furthermore, such patients frequently present excessive proliferation and activation of cholangiocytes – a phenomenon clinically known as “ductular reaction”. These observations are taken as evidence of aberrant or stalled regenerative processes during liver jury. It is therefore reasonable to hypothesize that better understanding of signaling that occurs in the liver stem cell niche during injury may be leveraged towards development of regeneration-correcting therapies.

What constitutes a liver stem/progenitor cell (LPC) niche? There is considerable debate in the liver biology community with regards to the location of liver stem cell niche, however, strong evidence points to Canals of Herring as being such a niche^5^,^6^. As shown in Fig. 1, Canals of Hering are located at the ductular–hepatocellular junction where putative LPCs are in close proximity to adult hepatocytes, cholangiocytes and periportal fibroblasts^7^,^8^. It is reasonable to presume that fate choices of LPCs are driven in part by the paracrine signals arriving from neighboring adult cells. These signals are not fully understood but include such morphogens as hepatocyte growth factor (HGF), Wnts, fibroblast growth factors (FGFs), hedgehogs and transforming growth factor (TGF)-β^9^,^10^,^11^,^12^,^13^,^14^,^15^,^16^. Of these, TGF-β1 is known to be a potent inducer of biliary epithelial (cholangiocytic) fate selection of stem cells whereas HGF promotes hepatic differentiation^8^,^9^,^11^,^14^,^16^. While a lot is known about inductive signals driving regenerative processes in the liver, their cellular origins are not well understood. In this study, we wanted to focus on a subset of cellular interactions likely occurring in the liver stem cell niche, those between adult hepatocytes and LPCs (see Fig. 1). We wanted to study these interactions in the context of alcohol injury.

Several groups, including ours, have demonstrated that microfluidic devices hold significant promise for cell cultivation and analysis^17^,^18^,^19^,^20^,^21^. Beyond well appreciated advantages of decreasing the need for cells and reagents, such devices elicit improved cell phenotype and function. This is being attributed to enhanced accumulation of endogenous growth factors and autocrine signals in confined volumes of microfluidic chambers operating under diffusion dominant transport conditions (low flow regime). Such enhanced autocrine signals have been observed in stem cells^18^,^22^,^23^, cancer cells^24^ and in primary hepatocytes^19^,^25^.

Recently, our group has demonstrated that hepatocytes engage in both autocrine and paracrine signaling inside multi-chamber microfluidic devices^19^. This past study revealed that hepatocytes produced sufficient amounts of HGF to affect phenotype of recipient cells located in a neighboring chamber several hundreds of micrometers away. In another recent study we employed multi-chamber microfluidic co-cultures of hepatocytes and stellate cells to study heterotypic interactions vis-a-vis TGF-β1 during alcohol injury^21^. This study revealed that alcohol injury triggered hepatic production of TGF-β1 which in turn caused stellate cells to become activated and begin producing TGF-β1 of their own. These past studies led us to hypothesize that injured hepatocytes may be a significant source of paracrine signals contributing to stem cell fate choices in the liver stem cell niche.

Primary LPCs are exceedingly challenging to isolate from animal or human tissue. In addition, these cells exhibit restricted expansion and differentiation in culture, lose *in vivo* phenotype, and display high variability from one isolation to the next^26^,^27^. Embryonic stem cells (ESCs), on the other hand, are readily expandable and may be differentiated into a desired cell type^28^. Several protocols for differentiating ESCs or iPSCs into hepatocytes have been reported in the literature^29^,^30^,^31^,^32^,^33^. Herein, we employed pluripotent stem cell technology to derive model LPCs - cells expressing early hepatic markers such as alpha fetal protein (AFP). Microfluidic co-culture devices were then used to place LPCs and primary hepatocytes into compartments separated by 100 μm long grooves. LPC differentiation in these microfluidic chambers was then investigated as a function of alcohol injury.

## Results and Discussion

### Design and operation of a microfluidic co-culture device

**Figure 1:**
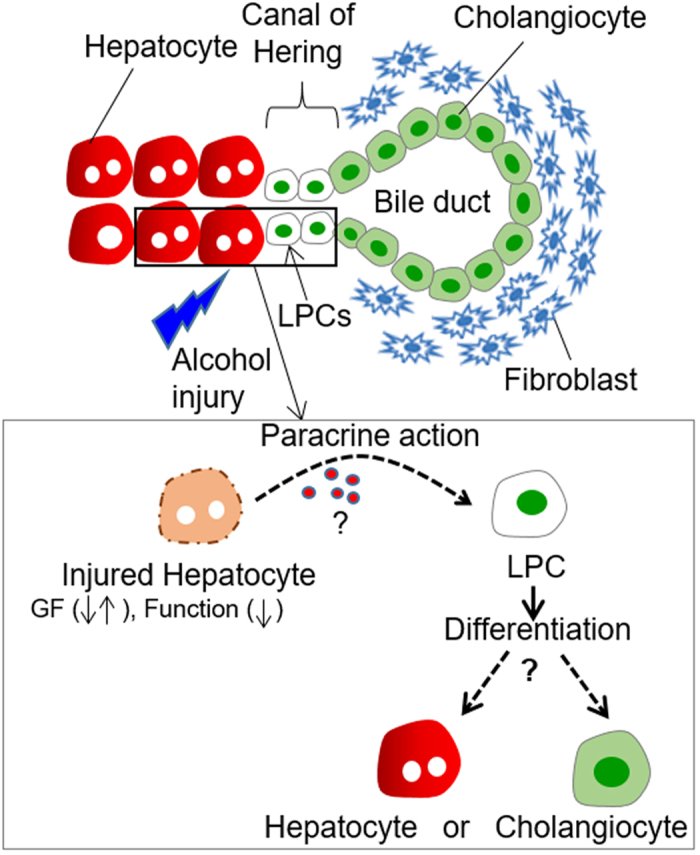
Putative liver stem cell niche in the Canals of Hering. Liver progenitor cells (LPCs) reside in close proximity to hepatocytes and cholangiocytes, and likely receive paracrine signals from neighboring adult cells during injury (top image). Our study explored heterotypic interactions between hepatocytes and LPCs, and tested a hypothesis that ethanol injury pushes LPC to choose cholangiocytic fate instead of the default hepatic fate (bottom image). GF, growth factor.

**Figure 2:**
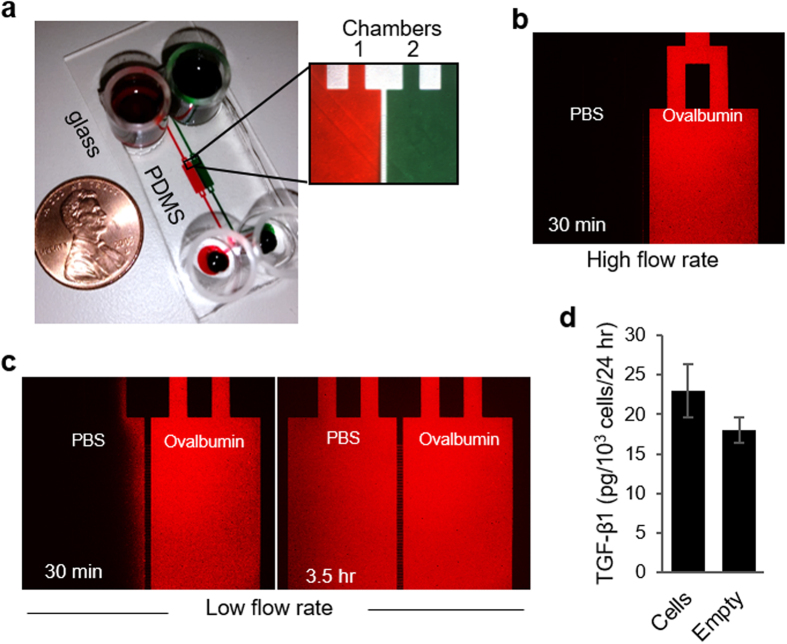
Layout and operation of the microfluidic co-culture device. (**a**) Two adjacent cell culture chambers filled with food dye for contrast. The chambers were connected by grooves to enable paracrine interactions. (**b**) Limited diffusion of ovalbumin-Alexa555 was observed between neighboring chambers in the high flow regime. (**c**) Diffusion of ovalbumin-Alexa555 between the chambers was enhanced under low flow regime. (**d**) The percent of TGF-β1 diffuse to the empty chamber was calculated from the total concentration of this growth factor measured in both chambers: empty chamber and the chamber occupied by hepatocytes (explained in method section). This pointed to unimpeded diffusion of paracrine signals.

A putative liver stem cell niche is located in the Canals of Hering, the junctional structure connecting bile canaliculi formed by hepatocytes with bile ducts lined by cholangiocytes^7^,^8^. Our objective was to employ microfluidic co-cultures chambers to recapitulate key heterotypic interactions occurring within the liver stem cell niche, those between adult hepatocytes and LPCs (Fig. 1). For this purpose, we designed a microfluidic platform comprised of two parallel cell culture chambers (1.8 mm in width, 5.6 mm in length and 75 μm in height) interconnected by grooves (5 μm in height, 20 μm in width and 100 μm in length) (Fig. 2a). Long and narrow channels (400 μm in width, 10 mm in length and 75 μm in height) were connecting cell culture chambers to inlet and outlet ports holding media (Fig. 2a). We have previously modeled transport in a microfluidic device of a similar geometry and have determined that glucose levels were comparable to standard large volume cultures^18^. The oxygen tension was expected to be significantly better inside microfluidic devices because of a short distance between the cells and the gas permeable PDMS membrane comprising the roof of the device.

The flow rate inside a microfluidic co-culture device could be high (~71 μl/h) or low (~0.4 μl/h) depending on media loading into the inlet/outlet ports. The high flow rate regime was used for selective seeding or retrieval of cells. To achieve this regime, media was only loaded into the inlet ports (cloning cylinders in Fig. 2a) while the outlet ports were empty initially. This created a transient high flow regime for ~30 min until liquid levels at the inlet and outlet equilibrated. As highlighted by microscopy images of fluorescently-labeled ovalbumin (Fig. 2b), limited diffusion between adjacent culture chambers occurred while the flow rate remained high. This fact could be leveraged to introduce proteases into one chamber in order to remove cells residing in this channel without affecting the cells in the neighboring chamber. Conversely, when liquid levels in the inlet and outlet were the same and flow rate was low (0.4 to 4 μl/h), diffusion across chamber was fairly rapid^34^. This is highlighted by images in Fig. 2c which show that 3.5 h after infusing fluorescent ovalbumin into one chamber, fluorescence in both chambers became similar. The results presented in Fig. 2c demonstrate that under correct flow conditions proteins can diffuse from one chamber to another and that paracrine communication was possible in our microfluidic device. Further evidence in support this was obtained by culturing hepatocytes in one microfluidic chamber while keeping the other chamber free of cells. TGF-β ELISA of media collected from each chamber revealed that the level of this GF was similar in the chamber with and without cells after 24 h of culture (Fig. 2d).

### Characterizing effects of alcohol injury on microfluidic hepatocyte cultures

**Figure 3:**
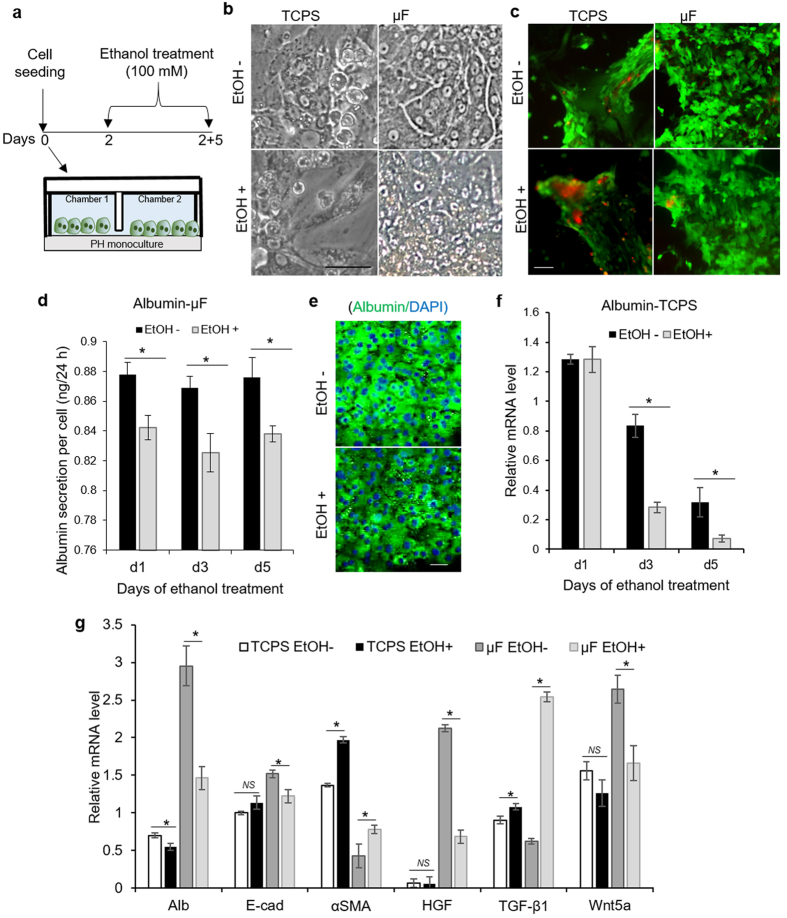
Response of hepatocyte to ethanol injury. Samples were analyzed on day 7 of culture (5 days of ethanol treatment) unless otherwise mentioned. (**a**) Diagram describing experiment where hepatocytes are exposed to 100 mM ethanol for 5 days. (**b**) Brightfield microscopy of hepatocytes cultured with and without ethanol showing mesenchymal phenotype in tissue culture polystyrene (TCPS) plates and epithelial phenotype in microfluidic (μF) devices. (**c**) Live (green) and dead (red) cell staining showing the effect of alcohol treatment on cell viability. (**d**) Albumin ELISA of healthy and injured hepatocytes cultured in microfluidic devices. (**e**) Immunofluorescent staining showing the expression of albumin in healthy and injured hepatocytes cultured in microfluidic devices. (**f**) Quantitative real-time PCR analysis reveals that the expression albumin in hepatocytes cultured in TCPS plates decrease rapidly even in the absence of injury by ethanol. (**g**) Real-time PCR analysis of hepatic marker (Alb), epithelial and mesenchymal markers (E-cad and αSMA), and growth factors (HGF, TGF-β1, and Wnt5a). The bars represent mean ± SD (n = 3). *P < 0.05. NS: non-significant. Scale bar: 50 μm. Abbreviation: Alb, albumin; E-cad, Epithelial-cadherin; αSMA, alpha smooth muscle action; HGF, hepatocyte growth factor; TGF-β1, transcription growth factor beta-1; Wnt5a, wingless-type MMTV integration site family, member 5a; PH, primary hepatocytes.

While the ultimate goal of this study was to investigate heterotypic interactions between progenitor cells and hepatocytes during alcohol injury, initial experiments were focused on understanding how hepatocytes alone respond to ethanol. In these experiments, we compared responses of primary hepatocytes cultured in standard culture dishes and in microfluidic channels to an insult with 100 mM ethanol. Cells were first cultured for 48 h in regular hepatocyte culture media and then treated with alcohol daily for next 5 days (Fig. 3a). In the absence of ethanol, hepatocytes cultured in microfluidic channels retained high levels of hepatic function (Fig. 3b). In the presence of 100 mM ethanol, these cells remained viable over the course of 5 days (Fig. 3c) but the markers of hepatic phenotype such as albumin were negatively affected by alcohol (Fig. 3d,e). The response of hepatocytes to alcohol insult in microfluidic cultures may be contrasted with standard large volume cultures (multi-well tissue culture plates). As seen from Fig. 3b,c,f, cell viability and function were decaying in a regular culture dish in the absence of ethanol but was further exacerbated by alcohol insult. This set of experiments established microfluidic devices as a better system for cultivation and injuring hepatocytes compared to standard multi-well plates.

Hepatocytes challenged with ethanol in microfluidic channels upregulated expression of mesenchymal marker alpha-smooth muscle actin (αSMA) and downregulated expression of epithelial markers, E-cadherin and albumin (Fig. 3g). Furthermore, ethanol injury was associated with upregulated expression TGF-β1 (4-fold) and downregulation of HGF (3-fold). HGF is the key morphogen known to drive liver development and regeneration processes. On the other hand, TGF-β1 is the main fibrogenic signal associated with loss of hepatic function and epithelial hepatic phenotype. The fact that expression of these key morphogens diverged upon ethanol injury to hepatocytes, with TGF-β coming up and HGF going down, may explain the change in phenotype from more epithelial to mesenchymal.

It is important to note that similar analysis carried out for hepatocytes maintained in tissue culture polystyrene (TCPS) plates did not reveal ethanol-induced changes in αSMA, albumin, E-cadherin, HGF or TGF-β1 (Fig. 3g). This may be explained by the fact that hepatocytes in standard culture dishes were de-differentiating rapidly even in the absence of injury. Taken together, our results confirm that microfluidic cultures of hepatocytes represent a better, more sensitive model of alcohol injury than standard large volume cell cultures.

Why are hepatocytes able to better tolerate alcohol insult in microfluidic cultures? Several reports, including from our group, have demonstrated that microfluidic channels may enhance cell function by helping to accumulate cell-secreted signals^18^,^22^,^23^. This was shown to be true for stem cells^18^, cancer cells^24^, and most recently for hepatocytes^34^. In the case of hepatocytes, the enhanced function was connected to accumulation and upregulation of HGF. Indeed, 30-times higher expression of HGF was observed for hepatocytes cultured in microfluidic channels compared to cells in standard 12-well plates (Fig. 3g). It is of note that HGF is known to have protective effects during alcohol injury^35^ and may aid in recovery from alcohol-induced fatty liver *in vivo*^36^,^37^,^38^,^39^. Therefore, it is reasonable to hypothesize that accumulation of protective endogenous signals such as HGF makes hepatocytes more tolerant to high levels of alcohol in microfluidic cultures.

In addition to HGF and TGF-β1, we characterized expression of other signals associated with differentiation of LPCs, including Wnt5a, driver of hepatic fate^13^, as well as Indian hedgehog (Ihh) and Sonic hedgehog (Shh)^10^ which are known to promote cholangiocytic fate. This analysis revealed that Wnt5a was negatively affected by ethanol (Fig. 3g) while the hedgehog signals were unaffected (data not shown). Based on these observations we hypothesized that healthy or injured hepatocytes may modulate the milieu of signals bathing the neighboring LPCs and may contribute to fate selection decisions of these cells.

### Enhanced hepatic differentiation of ESC-derived liver progenitor cells co-cultured with healthy hepatocytes

**Figure 4:**
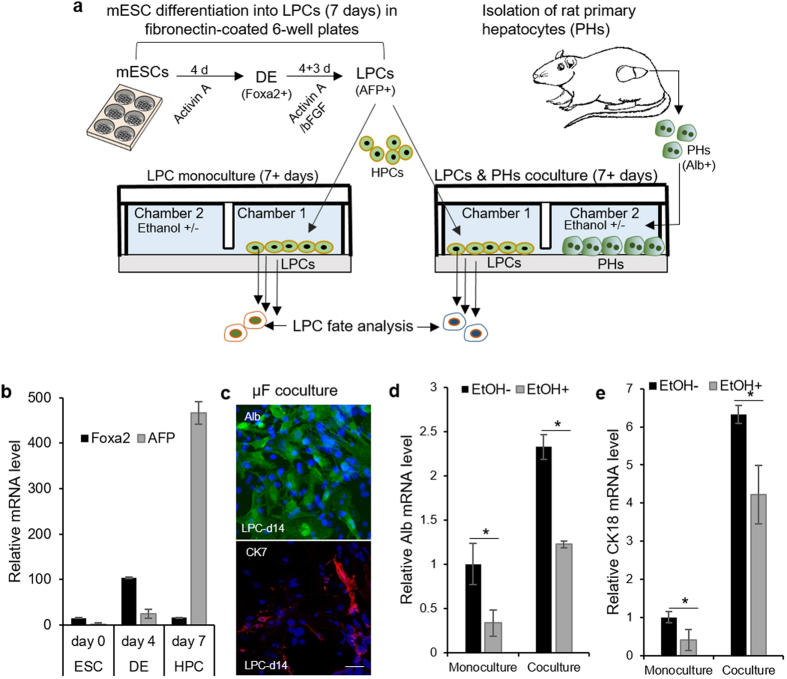
Response of mouse ESC-derived LPCs to functional hepatocytes in microfluidic cocultures. (**a**) Diagram showing experiment set-up where mESCs-derived LPCs (or HPCs) were differentiated under mono- and co-culture conditions in the presence and absence of 100 mM ethanol. (**b**) Quantitative real-time PCR analysis for mRNA expression of Foxa2 for definitive endoderm (DE) phase and alpha fetoprotein (AFP) for LPC phase. (**c**) Immunofluorescent analysis of albumin (Alb, green) and cytokeratin (CK)-7 (red) in LPCs on 7 days of coculture with functional hepatocytes. (**d,e**) RT-PCR expression analysis of hepatic markers (Alb and CK18) in LPCs differentiated under mono- and co-culture condition in the presence and absence of ethanol. LPC samples were analyzed on day 7 of co-culture with injured and functional hepatocytes. Monocultures of LPCs under the same condition were used as controls. The data indicate means ± SD (n = 3). Scale bar: 50 μm. *P < 0.05. All data are normalized to GAPDH.

Microfluidic co-cultures were used to test a hypothesis that hepatocytes may provide instructive paracrine signals to neighboring progenitor cells. Given that isolation of primary progenitor cells from the liver remains a challenge, we developed model LPCs by differentiating mESCs beyond definitive endoderm (DE) to the point where early hepatic markers were expressed. Stem cell differentiation protocol, shown in Fig. 4a, involved induction of DE by activin A for 4 days, followed by exposure to activin A and bFGF for 3 more days. DE stage was characterized by robust expression of Foxa2 (Fig. 4b) whereas LPCs exhibited decay in Foxa2 and increase in alpha fetal protein (AFP) (Fig. 4c). The differentiation protocol was carried out in a standard 6-well plate after which LPCs and primary hepatocytes were seeded separately into parallel microfluidic chambers.

Our first objective was to characterize interactions between healthy hepatocytes and LPCs. Immunostaining of LPCs after 7 days of differentiation in co-cultures revealed prominent expression of hepatic marker, albumin, while the expression of cholangiocytic marker, CK7, was not substantial (Fig. 4c). RT-PCR analysis confirmed immunostaining results, pointing to 2-fold increase in albumin expression and a 6-fold increase in cytokeratin (CK)-18, another hepatic marker, when compared to LPCs differentiated as monocultures (Fig. 4d,e). These results suggest that presence of paracrine signals from healthy hepatocytes enhances hepatic differentiation trajectory of neighboring LPCs.

### Alcohol injury to microfluidic co-cultures decreased hepatic and promoted cholangiocytic differentiation of mouse and human ESC-derived LPCs

In the next set of experiments microfluidic cultures of progenitor cells and hepatocytes were injured with 100 mM ethanol over the course of 7 days and were compared to uninjured cells (LPCs alone and together with hepatocytes). Ethanol administration diminished expression of hepatic markers, albumin and CK18, in LPC mono- and co-cultures (Fig. 4d,e). These results support our previous findings of ethanol treatment impairing hepatic differentiation of DE cells^40^. This impairment was shown to occur through inhibition of ERK signaling. It may also be noted from Fig. 4d,e, that hepatic marker expression in LPC-hepatocyte co-cultures during alcohol injury was higher than in LPC monocultures. This may be due to inductive paracrine signals originating from hepatocytes.

**Figure 5:**
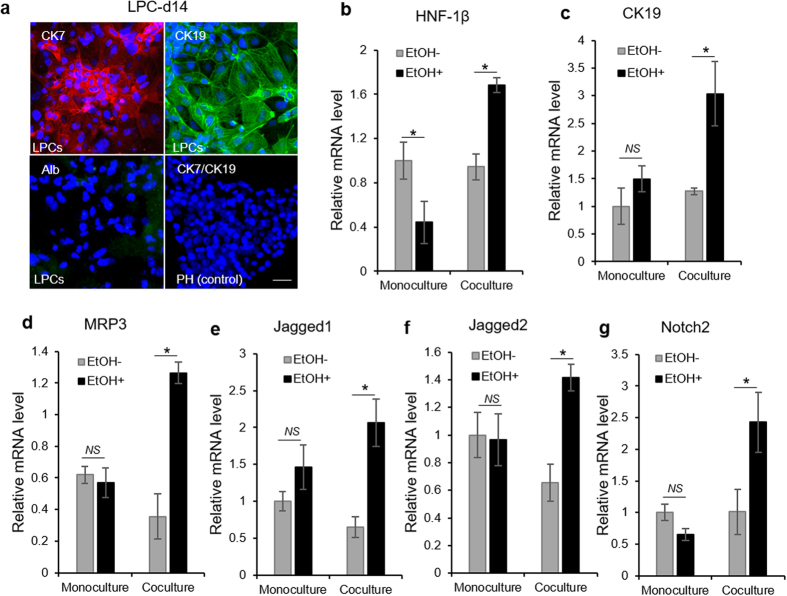
Fate selection of mouse ESC-derived LPCs during ethanol injury in a microfluidic device. (**a**) Immunostaining images showing prominent expression of cholangiocyte markers (CK7 and CK19), but not hepatic marker (albumin, Alb) in LPCs differentiated adjacent to injured hepatocytes for 7 days. Ethanol was introduced two days after cell seeding. Note that hepatocytes stained for neither marker (data not shown). Scale bar: 50 μm. (**b–g**) Real-time PCR analysis for mRNA expression level of cholangiocyte markers in LPCs differentiated for 8 days alone (monoculture) or next to hepatocytes (coculture) in the presence and absence of 100 mM ethanol. The data indicate means ± SD (n = 3). *P < 0.05. NS: non-significant. All data are normalized to GAPDH.

We also tested a hypothesis that alcohol injury may create a microenvironment conducive to cholangiocytic differentiation of LPCs. This hypothesis is based on recent reports connecting alcoholic hepatitis in humans with enhanced cholangiocytic differentiation of LPCs^41^. As seen from Fig. 5a, immunostaining analysis confirmed high levels of biliary markers (CK7 and CK19) in LPCs co-cultured with hepatocytes for 7 days during alcohol injury. At the same time minimal albumin expression was observed in these cells. Further gene expression analysis revealed that multiple biliary markers (CK19, MRP3, Jagged-1 and -2, HNF1β and Notch2) were upregulated in LPCs cultured next to hepatocytes during alcohol injury but not in LPCs injured in monocultures (Fig. 5b–g). Our observations using a microfluidic platform confirm *in vivo* results of alcohol exposure altering fate selection of LPCs in driving these cells towards biliary lineage instead of the default hepatic lineage^36^. It is worth noting that ethanol injury to LPC monocultures did not lead to a change in the expression of cholangiocyte markers, suggesting that alcohol treatment of LPC monocultures impairs hepatic differentiation but does not affect biliary differentiation. It appears that latter phenomenon requires presence of injured hepatocytes.

**Figure 6:**
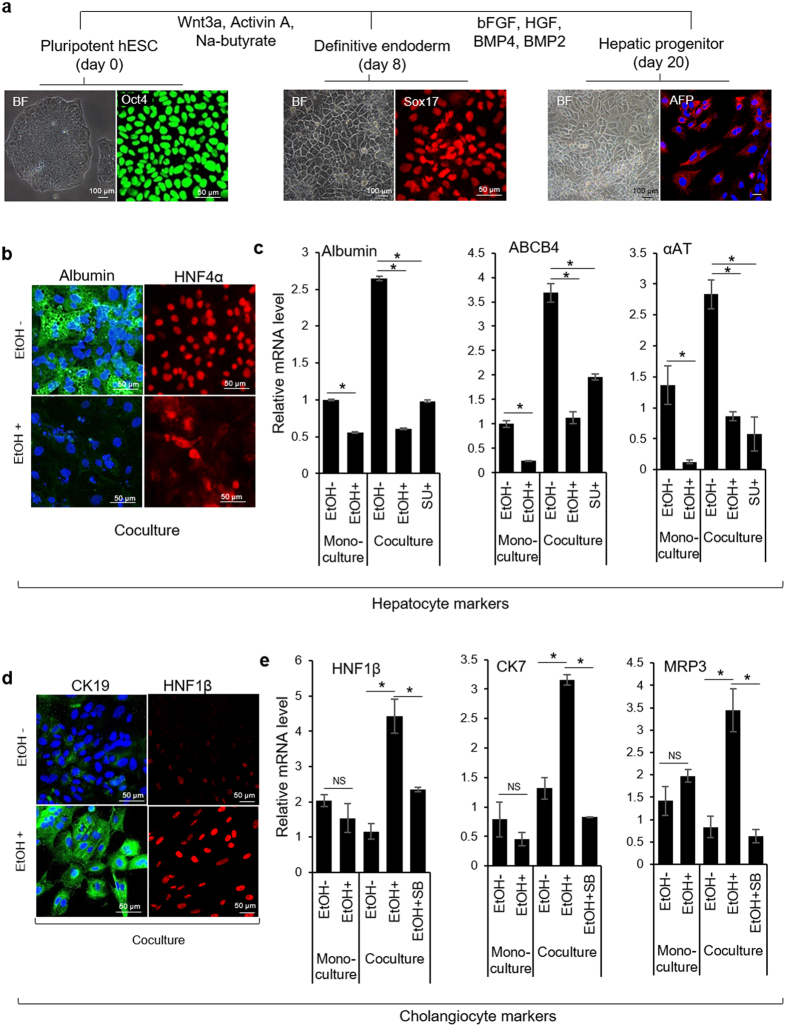
Response of human ESC-derived LPCs to ethanol insult in microfluidic devices. (**a**) Brightfield (BF) and immunostaining images showing the epithelial phenotype and expression of pluripotency marker (Oct4), definitive endoderm markers (Sox17), and liver/hepatic progenitor cell (LPC) marker (AFP). (**b**) Immunostaining images showing prominent expression of hepatic markers, albumin (green) and HNF4α (red) in LPCs cocultured with healthy primary hepatocytes for 7 days inside microfluidic chambers. (**c**) Real-time PCR shows that LPCs cultured next to healthy hepatocytes upregulated expression of hepatic markers (albumin, ABCB4, and αAT). The expression of hepatocyte markers was downregulated by small molecule inhibitor of HGF receptor (c-met) - SU11274. (**d**) Immunostaining showing expression of cholangiocyte markers, CK19 (green) and HNF1β (red) in LPCs cultured next to injured hepatocytes for 7 days. (**e**) qPCR analysis of cholangiocytic markers (HNF-1β, CK-7, and MRP3) in LPCs cultured next to ethanol-injured hepatocytes. Note that treatment with inhibitor of TGF-β1 signaling (SB431542) diminishes the influence of injured hepatocytes on cholangiocytic differentiation of LPCs. The data indicate means ± SD (n = 3). *P < 0.05. NS: non-significant. All data are normalized to GAPDH. Abbreviation: SU, SU11274; SB, SB431542; EtOH, ethanol, Na-butyrate, sodium butyrate.

While ESCs from different species exhibit similar differentiation trends, the inductive signals driving differentiation of mouse and human stem cells may be somewhat different. To investigate this point, we incorporated human ESC-derived LPCs into microfluidic co-cultures. For induction of DE, human ESC (Oct4+) on vitronectin were serum-starved in the presence of activin A and Wnt3a for 2 days, followed by treatment with activin A and sodium butyrate in serum containing media for another 6 days. To derive model LPCs, DE cells were cultured on collagen I in media containing HGF, FGF4, BMP2, and BMP4 for 12 days. DE and LPC phenotypes were characterized by robust expression of Sox17 and AFP, respectively (Fig. 6a). Similar to mouse ESCs, the differentiation of LPC was carried out in standard 6-well plates. LPCs and primary hepatocytes were then seeded separately in parallel microfluidic chambers for co-culture study. In order to understand the paracrine effect of HGF in directing LPC differentiation, we removed HGF from the differentiation media during microfluidic co-culture experiment.

Immunostaining of LPCs co-cultured with healthy hepatocytes for 7 days revealed prominent expression of hepatic markers, albumin and hepatocyte nuclear factor (HNF)-4α (Fig. 6b). In contrast, LPCs cultured next to injured hepatocytes were lacking in expression of hepatic markers, suggesting that hepatic differentiation was hampered by alcohol. Similar trends were observed by RT-PCR analysis which revealed a number of hepatic markers being upregulated in LPCs cultured next to healthy hepatocytes inside microfluidic devices. These markers included albumin, ATP-binding cassette transporter (ABCB4), and α-1-antityrpsin (αAT) (Fig. 6c). One should note that hepatic markers were expressed stronger in microfluidic co-cultures then in monocultures of progenitor cells in hepatic differentiation media. It is also interesting to note that alcohol injury to monocultures of human LPCs caused downregulation in the expression of hepatic markers in a manner similar to that observed for mouse LPCs (Fig. 4d,e).

Further analysis revealed that human LPCs co-cultured next to ethanol-injured hepatocytes expressed higher levels of biliary epithelial markers, CK19 and hepatocyte nuclear factor (HNF)-1β. CK19 showed an appropriate intracellular and reticular pattern (Fig. 6d), while HNF-1β showed nuclear localization, suggesting cholangiocytic differentiation in injured co-culture model. Gene expression analysis by RT-PCR confirmed upregulation of several other biliary markers (HNF1β, CK7, and multi-drug resistance protein-3 (MRP3)) in LPCs cultured next to hepatocytes during alcohol injury but not in LPCs injured in monocultures nor in co-cultures without ethanol (Fig. 6e). Overall, these results demonstrated that exposure to ethanol caused human progenitors cells to switch differentiation program from hepatic to cholangiocytic. Furthermore, we demonstrated that differentiation of progenitor cells inside microfluidic chambers is driven, at least in part, by the paracrine signals originating from neighboring hepatocytes and that composition of the milieu of signals is modulated by ethanol exposure. In the next set of experiments, we investigated involvement of two putative paracrine signals, HGF and TGF-β1, that were hypothesized to drive hepatic and cholangicoytic differentiation programs in progenitor cells.

### Interfering with hepatic HGF and TGF-β1 diminishes differentiation potential of LPCs

As discussed previously, HGF and TGF-β1 are the key inductive signals that direct hepatocyte and cholangiocyte differentiation programs, respectively. In Fig. 3g we demonstrated that cultivation of hepatocytes inside microfluidic devices resulted in upregulated expression of HGF when compared to standard multi-well plates. We also showed that exposure to alcohol in microfluidic devices caused 2.5-fold reduction in HGF expression, concomitant with 3-fold increase in TGF-β1 gene expression. To assess the involvement of HGF and TGF-β1 in shaping LPC fate in microfluidic co-cultures, we used small molecule inhibitors - SU11274 to block HGF receptor (c-met) and SB431542 to inhibit TGF-β signaling. As shown in Fig. 6c, inhibition of c-met in microfluidic co-cultures caused downregulation of hepatic markers in progenitor cells - albumin (2.5-fold), ABCB4 (2-fold), and αAT (6-fold) compared to LPCs cultured in the absence of inhibitor. Figure 6e demonstrates that addition of SB431542, a small molecule inhibiting signaling of TGF-β1 by interfering with activin receptor-like kinases (ALK4,5,7)^42^, diminished cholangiocytic differentiation of progenitor cells cultured next to injured hepatocytes. These results further confirm our hypothesis that endogenous growth factors, most likely of hepatic origin, control LPC fate selection in microfluidic co-cultures with hepatocytes.

## Conclusions

LPC fate selection is likely governed by the paracrine signals produced in the liver stem cell niche. The signals pushing LPCs along hepatic lineage include HGF and members of Wnt family, while cholangiocytic differentiation is promoted by TGF-β1, Hedgehog (Hh), Notch and other signals^9^,^10^,^11^,^12^,^13^,^14^,^15^,^16^. One putative stem cell niche is thought to be located in the Canals of Hering where LPCs are in close proximity to adult hepatocytes, adult cholangiocytes, and portal fibroblasts^8^. In this paper we sought to recapitulate one type of heterotypic interactions occurring within the stem cell niche, those between adult hepatocytes and LPCs. Furthermore, we were interested in investigating the effects of alcohol injury on fate selection of LPCs. To achieve this goal, we implemented a microfluidic co-culture platform. This platform allowed to harness endogenous signals (e.g. HGF), offering a simple yet effective means of maintaining primary hepatocytes in a differentiated state over multiple days in culture. Importantly multi-day exposure to high levels of alcohol did not compromise viability of hepatocytes. It did however result in lower expression of hepatic markers (e.g. albumin), downregulation of hepato-inductive signals (HGF, Wnt5a) and upregulation of profibrogenic signal TGF-β1. These experiments established microfluidic cultures as a promising model of alcohol injury to hepatocytes. In subsequent experiments, injured or healthy hepatocytes were co-cultured with LPCs in order to characterize stem cell fate decisions as a function of injury. These experiments clearly demonstrated that exposure to alcohol in co-cultures with hepatocytes drove LPCs towards cholangiocytic fate while injury to LPC monocultures simply impeded differentiation. In the absence of alcohol insult, LPCs co-cultured with hepatocytes differentiated along hepatic lineage. Importantly, inhibition of HGF and TGF-β signaling in cocultures abrogated hepatic and cholangiocytic differentiation of LPCs, respectively.

**Figure 7:**
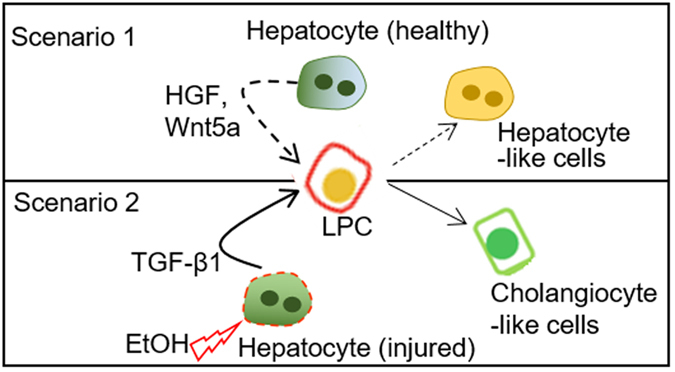
Proposed mechanism of progenitor cell fate decisions in the context of ethanol injury. In the absence of ethanol, paracrine signals from healthy hepatocytes, including HGF, drive hepatic differentiation of progenitor cells (scenario 1). Ethanol injury causes hepatocytes to downregulate production of HGF while upregulating TGF-β1 (scenario 2). The growth factors secreted by functional rat primary hepatocytes enhance the maturation of ESC-derived liver progenitor cells (LPCs) into hepatic lineage (scenario 1), while alcohol treatment of LPCs co-cultured with adult liver cells enhances the differentiation towards the cholangiocyte fate possibly by the up-regulation of TGF-β1 (scenario 2).

Is it physiological to expose hepatocytes and LPCs to alcohol together? It is highly likely that LPCs are exposed to alcohol *in vivo* since they reside in close proximity to the hepatocyte plate^43^. Therefore, we believe that simultaneous exposure of hepatocytes and LPCs to alcohol accurately reflects physiology of alcohol injury *in vivo*. The findings presented in this paper are interesting for several reasons. First, we identify microfluidic cell cultures as a promising *in vitro* model of alcohol injury where primary hepatocytes remain highly functional in the presence of high concentration of ethanol. Our *in vitro* model may be interesting in light of complexity and limitations of animal models of alcohol injury as well as difficulties in parsing out cellular crosstalk and signaling in such models. Second, we demonstrate that microfluidic culture systems help accumulate endogenous growth factors produced by hepatocytes. The high levels of endogenous signals such as HGF contribute to maintenance of hepatic phenotype in microfluidic cultures. Third, we highlight the fact that alcohol injury modulates levels of hepatic HGF, Wnt5a and TGF-β1 and that these signals may act in a paracrine manner to drive fate decisions of neighboring LPCs (see proposed model described in Fig. 7). Thus, a microfluidic platform not only enhances autocrine signaling but enables paracrine interactions between liver cell types. Finally, we provide evidence that our microfluidic platform recapitulates aspects of ductular reactions – a clinical phenomenon observed in patients with chronic liver injury including alcoholic hepatitis^4^. The ability to recapitulate aspects of disease pathophysiology is an important step towards positioning microfabricated cell cultures as viable and valuable disease models.

Moving forward, the microfluidic platform may be made more complex through incorporation of additional cell types present in the Canals of Hering (e.g. fibroblasts and/or adult cholangiocytes). Such cultures may be also equipped with biosensors for monitoring local concentrations and gradients of secreted signals^21^. Furthermore, microfluidic cultures may be modified to include hydrogel ligand traps of the type shown by us recently^24^ to knock down specific paracrine signals originating from the desired cell type. In the future, microfluidic devices may be used to elucidate dynamics, mechanisms and cellular origins of pathogenic signaling and may be leveraged towards developing novel therapeutic strategies.

## Materials and Methods

### Fabrication of microfluidic co-culture devices

Microfluidic devices were fabricated by soft lithography approaches reported by us previously^44^. Briefly, master mold was fabricated by patterning SU-8 (Microchem Corp) on silicon wafer (University Wafer) in two steps. First, the 5 μm tall height and 100 μm long grooves connecting cell culture chambers were patterned by UV exposure of SU-8 (2005) and through an appropriate photomask (CAD/Art Services). Subsequently, the second transparent mask and the negative photoresist (SU-8 2050) were used for patterning the 75 μm tall rectangular cell culture chambers in registration with communication grooves described above. The polydimethylsiloxane (PDMS; Dow Corning) layer was made by pouring a mixture of 10:1 ratio of the base to curing agent onto the master SU-8 mold and baking at 70 °C for 80 min following a 30 min degassing step. The single PDMS layer was removed from the mold upon solidification and four holes were created using a sharp metal punch for generating segregated inlets and outlets for the parallel cell culture channels. In order to prevent leakage, single PDMS layer was irreversibly bonded to a 75 mm × 25 mm glass slide using oxygen plasma treatment. Glass cloning cylinders (10 mm; Fisher Scientific) were then glued atop the inlets and outlets in order to serve as the media reservoirs. Typical co-culture platform is shown in Fig. 2a.

The predictions regarding diffusion of secreted factors were experimentally verified by using a fluorescently-labeled protein, ovalbumin-Alexa Fluor 555 conjugate (Thermo Fisher). Fluorescently labeled ovalbumin was infused in one chambers and its diffusion to the adjacent chamber was assessed over the period of time. The high flow regime was achieved by loading 200 μl of labeled ovalbumin and PBS into the inlets, while equal volume (200 μl) of ovalbumin and PBS were added into the inlets and outlets to create a low flow regime. Flow rates inside microfluidic devices were measured by monitoring movement of beads, which has been explained elsewhere^34^.

### Culture and differentiation of mouse ESCs into liver progenitor cells (mLPCs)

Mouse ES-D3 [D3](ATCC CRL-1934) were propagated on growth-arrested mouse embryonic fibroblasts (CF-1 MEF, GlobalStem) in ES FBS medium consisting of DMEM supplemented with 15% ES FBS (Invitrogen), 2 mM L-glutamine, 1 mM MEM NEAA, 100 nM 2-mercaptoethanol, 1 × penicillin-streptomycin, and 10^3^ U/mL LIF (ESGRO, Millipore). Undifferentiated mouse ESCs were kept at 37 °C, 5% CO_2_ and 90–95% humidity, with medium exchanged daily, and passaged every 3 days using trypsin-EDTA. Prior to differentiation, cells were cultured in 6-well tissue culture plates pre-coated with 0.1 mg/ml fibronectin for 24 hrs in ES FBS media. To initiate differentiation, cells were switched to endoderm differentiation media containing IMDM (Gibco) supplemented with 10% ES-FBS, 2 mM L-glutamine, 1 mM MEM NEAA, 100 nM 2-mercaptoethanol, 1 × penicillin-streptomycin, and 50 ng/ml Activin A (Invitrogen) for 4 days to generate definite endoderm (DE) cells. From day 5 through day 7 DE cells were cultured in the same differentiation media together with 50 ng/ml basic fibroblast growth factors (bFGF) to induce DE cells into mLPCs. Fresh media was exchanged every two days throughout the differentiation experiments.

### Culture and differentiation of human ESCs into liver progenitor cells (hLPCs)

All human ESC studies used a federally approved human ESC line from Rockefeller University (RUES2, NIH Stem Registry identification No. 0013). Undifferentiated RUESC2 hESCs were maintained under feeder-free conditions, using vitronectin-coated plates and fed daily with E8 media (Invitrogen)^45^. hLPC differentiation started from the induction of definitive endoderm (DE) from hESCs using previously described protocols^46^. Briefly, undifferentiated hESCs on vitronectin were induced to DE in RPMI 1640 medium (Invitrogen) containing 100 ng/ml activin A (Peprotech), 50 ng/ml Wnt3a (R&D Systems) and 1% (vol/vol) penicillin/streptomycin (Gibco) for 2 days and then the medium was changed to RPMI 1640 medium with 100 ng/ml activin A, 0.5 mM sodium butyrate and 1 × B27 supplement (Invitrogen) for 6 days. DE cells were then split and re-seeded on collagen I (BD Bioscience)-coated 6-well plates in IMDM media (Invitrogen) supplemented with 20% FBS (Invitrogen), FGF-4 (20 ng/ml, Invitrogen), HGF (20 ng/ml, Invitrogen), BMP2 (10 ng/ml, Invitrogen), BMP4 (10 ng/ml, Invitrogen), 0.3 mM 1-thioglycerol (Sigma), 100 nM dexamethasone (Sigma) and 0.126 U/ml human insulin (Novolin N) for 12 days (total 20 days of differentiation). 0.5% DMSO (Sigma) was added in the differentiation media one day after seeding onto collagen-coated plates.

### Co-culture of primary hepatocytes and LPCs

All animal experiments were performed with the approval of the IACUC (Institutional Animal Care and Use Committee) of UC Davis (University of California, Davis) and in accordance with the Ethical Guidelines for Animal Experimentation of UC Davis. All adult female Lewis rats were purchased from Charles River Laboratories (Boston, MA). Isolation of primary rat hepatocytes was carried out as described in our previous publications^35^. On average, around 100 million primary rat hepatocytes with above 90% viability (confirmed by Trypan Blue) were isolated. As described previously^47^ DMEM media from Gibco (Dulbecco’s Modified Eagle Medium) supplemented with 1% (v/v) penicillin-streptomycin (Invitrogen), 10% (v/v) Fetal bovine serum (Invitrogen), 0.5 U/ml Insulin (Novolin N), 7 ng/ml glucagon (Sigma), 7.5 μg/ml hydrocortisone sodium succinate (Sigma) and 20 ng/ml EGF (Invitrogen) was used to culture primary rat hepatocytes.

For microfluidic co-cultures, the designated channel for primary rat hepatocytes was coated with 0.2 mg/ml of collagen type I, whereas the parallel channel was coated with 0.1 mg/ml of fibronectin (Millipore) for mLPCs and 0.2 mg/ml collagen type I for hLPCs. Prior to cell seeding, each chamber was washed once with PBS and once with culture medium. For the purposes of uniform seeding in microchambers, the cell suspension was maintained at a density of 4 × 10^6^ cells/ml for primary hepatocytes and 2 × 10^6^ cells/ml for LPCs. 50 μl of hepatocyte and LPC suspensions were added separately in the inlets. The convention driven flow due to the high pressure differences at inlets and outlets allowed the cells to reach into the cell culture chambers and prevented mixing of the two cell types (explained in result section). This resulted in seeding density between 7,000–10,000 cells for hepatocytes and 5,000–8,000 cells for LPCs. After seeding, 250 μl of hepatocyte culture medium was added in the inlet and outlet for the chambers seeded with primary hepatocytes, whereas hepatic differentiation media was used for the adjacent channel to culture LPCs. As a control, identical coculture experiment was performed except that primary hepatocytes were not seeded adjacnet to LPCs. Moreover, microscope glass slides (1.2 cm × 1.2 cm) in 12-well tissue culture plates also served as controls for the conventional tissue culture platform. Glass slides were coated with either 0.1 mg/ml of fibronectin solution for mLPCs and with 0.2 mg/ml of collagen type I for hLPCs and primary hepatocytes. For seeding on coated glass slides in 12-well tissue culture plates, primary rat hepatocytes were suspended in media at a concentration of 100,000 cells/cm^2^ and stem cells at a concentration of 80,000 cells/cm^2^ and incubated for 1–2 hr at 37 °C. Unattached cells were removed by one-time wash with culture media. Culture medium was changed in all samples every 48 hours. Because of the fact that human ESCs are more dependent on growth factors than mESCs for hepatic differentiation^48^, complete growth factor-free and HGF-free differentiation media were used for mLPCs and hLPCs, respectively. These differentiation media were used to monitor the effect growth factors released by functional and injured hepatocytes.

For injury experiment, ethanol was used in hepatocyte culture media to treat the monoculture of primary hepatocytes as well as co-culture of primary hepatocytes and LPCs. Cells were seeded in regular tissue culture plates and microfluidic chambers 2 days prior to ethanol treatment. 100 mM of ethanol was used to treat monocultures and co-cultures daily for 5 days. Similar to ethanol treatment, HGF receptor (c-met) inhibitor (SU11274, 5 μM, Sigma) and TGF-β1/ALK receptor inhibitor (SB431542, Sigma) was utilized for 5 days where indicated.

### ELISA

Albumin secretion was assessed using commercial ELISA assay (Bethyl Laboratories). TGF-β1 secretion from primary rat hepatocytes was assessed using commercial ELISA kit (R&D systems). Albumin and TGF-β1 values were normalized to the cell number. ImageJ was used to count the cell number from DAPI stained images. The theoretical prediction of GF diffusion between culture chambers of co-culture device was experimentally verified by TGF-β1 ELISA (see Fig. 2d). Hepatocytes were first cultured in one chamber of microfluidic co-culture device for 7 days with daily media exchange. The neighboring culture chamber was kept empty throughout the culture period. On day 7 media was collected separately from both of the culture chambers (empty and occupied) and the levels of TGF-β1 produced by the hepatocytes over the last 24 hours was measured by ELISA. The percent of TGF-β1 diffuse to the empty chamber was calculated from the total concentration of this growth factor measured in both chambers (empty chamber and the chamber occupied by hepatocytes).

### Live-dead cell imaging

Primary hepatocyte samples for live/dead cell analysis were stained using manufacturer’s instructions (Live/Dead cell viability kit, Life Technologies). Briefly, cells were washed with PBS prior to the treatment with PBS containing 4 μM ethidium homodimer and 2 μM calcein AM for 30 min at 37 °C. The samples were then rinsed with fresh PBS before being imaged laser scanning confocal microscope (LSM700, Carl Zeiss, Jena, Germany).

### Immunostaining analysis

Prior to fixing, all samples were washed thoroughly once with PBS. Both the primary rat hepatocytes and stem cells were fixed by the fixate solution containing 4% paraformaldehyde (Electron Microscopy sciences) and 0.2% Triton-X100 (Invitrogen) in PBS. Samples were incubated with the fixate solution for 15 min in room temperature, and subsequently washed 3 time with PBS afterwards. Furthermore, all samples were blocked for 1 h in room temperature with the 1% BSA solution (1% bovine serum albumin in PBS) prior to Immunostaining and then washed 3 times with PBS. All primary and secondary antibodies were diluted in the blocking solution (1% BSA) prior to incubation with samples. All samples were incubated for 90 min with the primary antibody solutions and 60 min with DAPI (Invitrogen) and the secondary antibody solutions at room temperature. Samples were washed 3 times with PBS after each incubation step. The primary antibodies used were: sheep anti-rat albumin (1:100; Bethyl lab Inc.), rabbit anti-CK19 (1:100, Santa Cruz biotechnology), mouse anti-CK7 (1:100, Millipore), rabbit anti-Oct4 (1:100, Santa Cruz biotechnology), goat anti-Sox17 (1:250, R&D), goat anti-AFP (1:100, Santa Cruz biotechnology), goat anti-albumin (1:100, Bethyl lab Inc.), rabbit HNF-1β (1:100, Santa Cruz biotechnology), rabbit HNF-4α (1:100, Santa Cruz biotechnology). All secondary antibodies and DAPI were diluted 1:1000. The secondary antibodies used were: Alexa-488 donkey anti-sheep IgG, Alexa-488 donkey anti-rabbit IgG, Alexa-546 donkey anti-rabbit IgG, Alexa-546 donkey anti-mouse IgG, Alexa-546 donkey anti-rabbit IgG, Alexa-488 donkey anti-goat IgG, and Alexa-555 donkey anti-rabbit IgG. All samples were imaged using a laser scanning confocal microscope (LSM700, Carl Zeiss, Jena, Germany).

### Quantitative real-time PCR

Protocol for collecting cells was similar to the one described for cell seeding. Loading of uneven liquid levels in the inlet and outlet was used to create convection dominated flow with minimal diffusive transport between the culture chambers. 100 μl of 0.05% trypsin-EDTA (Gibco) was added in the inlet of the stem cell channel, whereas hepatocytes channel was treated with 100 μl of PBS. After 5 mins of incubation at 37 °C, dissociated LPCs were collected at their designated outlet, while hepatocytes remained confined in the parallel channel. Similar approach was used to retrieve primary hepatocytes. For PCR analysis, total RNA was isolated using the High Pure RNA Isolation Kit (Roche), according to manufacturer’s instructions. RNA was converted to cDNA using the Transcriptor First Strand cDNA synthesis kit (Roche), and the quantitative PCR reactions were set up using the FastStart Universal SYBR Green Master (Roche. All PCR reactions were done in duplicate. Primers are listed in Table S1. The relative expression level of each gene was calculated using the comparative threshold cycle (Ct) method with GAPDH as a housekeeping gene.

### Statistical analysis

All experiments were performed with both technical and biological duplicates. Standard deviation (SD) for the biological duplicates were presented as the error bars. Statistically significant difference was determined through student t-test at p < 0.05.

## Additional Information

**How to cite this article**: Haque, A. *et al*. Ductular reaction-on-a-chip: Microfluidic co-cultures to study stem cell fate selection during liver injury. *Sci. Rep.* **6**, 36077; doi: 10.1038/srep36077 (2016).

**Publisher’s note:** Springer Nature remains neutral with regard to jurisdictional claims in published maps and institutional affiliations.

## Supplementary Material

Supplementary Information
