# Supplementary material for: Ductular reaction-on-a-chip: Microfluidic co-cultures to study stem cell fate selection during liver injury
Source: Sci Rep. 2016 Oct 31;6:36077. doi: 10.1038/srep36077 (PMC5086854; doi:10.1038/srep36077)
Supplement: Supplementary Information [file srep36077-s1.pdf]

SUPPLEMENTARY INFORMATION

Ductular reaction-on-a-chip: Microfluidic co-cultures to study stem cell fate selection during liver injury

Amranul Haque[1, #] , Pantea Gheibi[1, #], Gulnaz Stybayeva[1], Yandong Gao[1], Natalie Torok,[2] Alexander Revzin[1]*

Supplementary Table 1: Primers used for transcript quantification by Real Time PCR

Human Albumin-F	GCACAGAATCCTTGGTGAACAG
Human Albumin-R	ATGGAAGGTGAATGTTTTCAGCA
Human αAT-F	ACTGTCAACTTCGGGGACAC
Human αAT-R	CATGCCTAAACGCTTCATCA
Human ABCB4-F	CTTTTCCTTGTCGCTGCTAAAT
Human ABCB4-R	AGTTCAGTGGTGTCGTTGATGT
Human Hnf1β(a)-F	GTACGTCAGAAAGCAACGAGAGAT
Human Hnf1β(a)-R	TGACTGCTTTTGTCTGTCATATTTCCA
Human CK7-F	AGACGGAGTTGACAGAGCTG
Human CK7-R	GGATGGCCCGGTTCATCTC
Human MRP3-F	AAAAGCAGACGGCACGACA
Human MRP3-R	GCAGGCACTGATGAGGAAGC
Human GAPDH-F	GGTGGTCTCCTCTGACTTCAACA
Human GAPDH-R	GTGGTCGTTGAGGGCAATG
Rat Albumin-F	CATCCTGAACCGTCTGTGTG
Rat Albumin-R	TTTCCACCAAGGACCCACTA
Rat HGF-F	CTTCTGCCGGTCCTGTTG
Rat HGF-R	TCTTCTCTTCTTCTGTCCTTCTGC
Rat TGF-β-F	CCTGGAAAGGGCTCAACAC
Rat TGF-β-R	CAGTTCTTCTCTGTGGAGCTGA
Rat E-cadherin-F	CGTGGATGTGGTAGACGTGAA
Rat E-cadherin-R	TTCTTCGCAGGCACAAAAAT

Rat Wnt5a-F	AGCCGAGAGACAGCCTTCAC
Rat Wnt5a-R	TCCTGCGACCTGCTTCATTG
Rat SMA-F	TGCCATGTATGTGGCTATTCA
Rat SMA-R	ACCAGTTGTACGTCCAGAAGC
Rat GAPDH-F	AGACAGCCGCATCTTCTTGT
Rat GAPDH-R	CTTGCCGTGGGTAGAGTCAT
Mouse Foxa2-F	GTCGTCCGAGCAGCAACATC
Mouse Foxa2-R	GGGTAGTGCATGACCTGTTCG
Mouse AFP-F	AAAGCTGCGCTCTCTACCAG
Mouse AFP-R	GAGTTCACAGGGCTTGCTTC
Mouse Albumin-F	AGTGTTGTGCAGAGGCTGAC
Mouse Albumin-R	TTCTCCTTCACACCATCAAGC
Mouse CK19-F	TGACCTGGAGATGCAGATTG
Mouse CK19-R	CCTCAGGGCAGTAATTTCCTC
Mouse HNF1β-F	AGAGCCCAGGCAGTCACA
Mouse HNF1β-R	GGGGTTCCTGCTTATGTGC
Mouse Notch2-F	TGCCTGTTTGACAACTTTGAGT
Mouse Notch2-R	GTGGTCTGCACAGTATTTGTCAT
Mouse Jagged1-F	GAGGCGTCCTCTGAAAAACA
Mouse Jagged1-R	ACCCAAGCCACTGTTAAGACA
Mouse Jagged2-F	CGTCATTCCCTTTCAGTTCG
Mouse Jagged2-R	CCTCATCTGGAGTGGTGTCA
Mouse MRP3-F	ACCAGGAGGACCATGAAGC
Mouse MRP3-R	TGTGGGTGCTGAGTGTGTCT
Mouse CK18-F	CAAGGTGAAGAGCCTGGAAA
Mouse CK18-R	AAGTCATCGGCGGCAAG
Mouse GAPDH-F	CCCCAATGTGTCCGTCGTG
Mouse GAPDH-R	GCCTGCTTCACCACCTTCT
